# Solid Lipid Nanoparticles Carrying Temozolomide for Melanoma Treatment. Preliminary In Vitro and In Vivo Studies

**DOI:** 10.3390/ijms19020255

**Published:** 2018-01-24

**Authors:** Nausicaa Clemente, Benedetta Ferrara, Casimiro Luca Gigliotti, Elena Boggio, Maria Teresa Capucchio, Elena Biasibetti, Davide Schiffer, Marta Mellai, Laura Annovazzi, Luigi Cangemi, Elisabetta Muntoni, Gianluca Miglio, Umberto Dianzani, Luigi Battaglia, Chiara Dianzani

**Affiliations:** 1 Dipartimento di Scienze della Salute, Università del Piemonte Orientale, Via Solaroli, 17, 28100 Novara, Italy; nausicaa.clemente@med.uniupo.it (N.C.); luca.gigliotti@med.uniupo.it (C.L.G.); elena.boggio@med.uniupo.it (E.B.); umberto.dianzani@med.uniupo.it (U.D.); 2 Dipartimento di Scienza e Tecnologia del Farmaco, Università degli Studi di Torino, Via Pietro Giuria 9, 10124 Torino, Italy; benedetta.ferrara@unito.it (B.F.); luigi.cangemi@unito.it (L.C.); elisabetta.muntoni@unito.it (E.M.); gianluca.miglio@unito.it (G.M.); chiara.dianzani@unito.it (C.D.); 3 Dipartimento di Scienze Veterinarie, Università degli Studi di Torino, Largo Paolo Braccini 2, 10095 Grugliasco (TO), Italy; mariateresa.capucchio@unito.it (M.T.C.); elena.biasibetti@unito.it (E.B.); 4 Centro di Neuro Bio Oncologia, Policlinico di Monza, Via Pietro Micca 5, 13100 Vercelli, Italy; davide.schiffer@unito.it (D.S.); marta.mellai@cnbo.it (M.M.); laura.annovazzi@cnbo.it (L.A.)

**Keywords:** SLN, melanoma, temozolomide

## Abstract

Aim: To develop an innovative delivery system for temozolomide (TMZ) in solid lipid nanoparticles (SLN), which has been preliminarily investigated for the treatment of melanoma. Materials and Methods: SLN-TMZ was obtained through fatty acid coacervation. Its pharmacological effects were assessed and compared with free TMZ in in vitro and in vivo models of melanoma and glioblastoma. Results: Compared to the standard free TMZ, SLN-TMZ exerted larger effects, when cell proliferation of melanoma cells, and neoangiogeneis were evaluated. SLN-TMZ also inhibited growth and vascularization of B16-F10 melanoma in C57/BL6 mice, without apparent toxic effects. Conclusion: SLN could be a promising strategy for the delivery of TMZ, allowing an increased stability of the drug and thereby its employment in the treatment of aggressive malignacies.

## 1. Introduction

Temozolomide (TMZ) is an imidazotetrazine anticancer drug (194 MW) endowed with an interesting profile. The presence of three adjacent nitrogen atoms in the heterocyclic ring, confers to this compound unique physical-chemical properties, as well as a remarkable antitumour activity [1]. 

**Figure 1:**
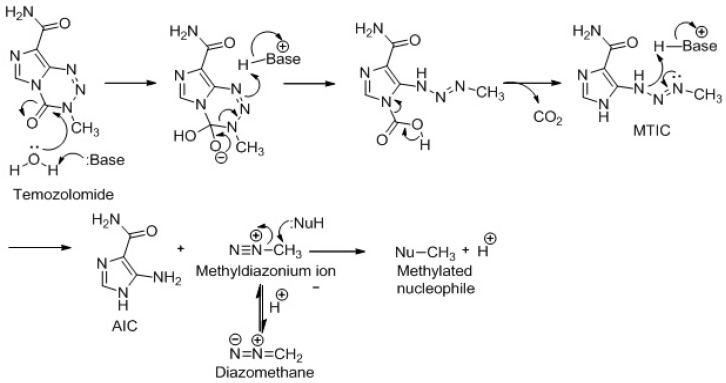
Scheme of TMZ ring opening. methydiazoniumion

TMZ is not affected by the acid pH of the stomach and is rapidly absorbed by the gastro-intestinal tract. However, in the blood and other tissues, it is first hydrolyzed to 5-(3-dimethyl-1-triazenyl) imidazole-4-carboxamide (MTIC), which is then quickly converted into its reactive form, the methyl-diazonium ion [2]. The methyldiazonium ion formed by the breakdown of MTIC primarily methylates guanine residues in the DNA molecule, resulting in the formation of O6- and N7-methylguanines (Figure 1). Initially, it was speculated that the first step of this activation process could be promoted by the specific local microenvironment conditions found in the major loop of the DNA helix. However, this hypothesis was difficult to be confirmed, and now it is believed that TMZ turns into MTIC even in in the absence of DNA [3,4,5]. Furthermore, the similarity between the half-life of TMZ in phosphate buffer pH 7.4 and in plasma from patients undergoing i.v. or oral administration indicates that the transformation TMZ → MTIC is a spontaneous and pH-dependent reaction, which occurs without the involvement of any catalysis by enzymes or other macromolecules [6]. The absence of any hepatic involvement in the activation process of TMZ may contribute to its high reproducibility pharmacokinetics, regardless of interpersonal variations in hepatic conversion rate [7].

The spontaneous and rapid activation, is however an unwanted change of this drug, with relevant consequences. Indeed, an intense dosage is required to gain therapeutic efficacy of TMZ. Moreover, it has been associated to the accumulation of MTIC in off-target tissues, and the occurrence of several side effects, such as myelosuppression, liver, heart and pulmonary toxicity [8].

The development of resistance to TMZ, that can decrease its efficacy towards tumour cells, can also affect the therapeutic potential of this agent. The O6-alkylguanine-trasferase (AGT) repairing enzyme and the mismatch repair system (MMR) are involved in the molecular processes underlying resistance to TMZ Moreover, it is well known that patients with hyper-methylated AGT promoter are more sensitive to TMZ chemotherapy; it is also reported, however, that TMZ is able to deplete the levels of AGT in various cell types, thus reducing the potential for drug resistance [9]. The ability of TMZ to cross the blood-brain barrier (BBB) allows its use as an agent for the adjuvant chemotherapy of glioblastoma (GB). Moreover, despite the fact that melanoma often tends to be resistant to TMZ and consequently a poor response rate is chemotherapy, due to its ease of use and high bioavailability, it can be employed in place of dacarbazine as the second line chemotherapy for this type of malignancies [10,11].

In a previous study, TMZ esters with short chain alcohols were synthesized, aiming to topical melanoma treatment. The median inhibitory concentration (IC_50_) of the esters was comparable to that of the standard TMZ, but the esters showed increased activity, because of improved skin penetration [12]. However, owing to the stage of the disease, treatment of advanced/metastatic melanoma often needs systemic administration of chemotherapeutic compounds [13]. Therefore, the aim of this study was to develop novel nanoparticulate delivery system for TMZ suitable for i.v. administration, in order to overcome its intrinsic drawbacks, and improve its therapeutic efficacy. Among nanoparticles, solid lipid nanoparticles (SLN) proved to be safe, biocompatible and effective to encapsulate drugs in order to ameliorate their ability to cross biological barriers, increase their stability in the biological environment, and overcome drug resistance [14].

In this experimental study an optimized formulation was obtained, through the fatty acid coacervation method [15], in order to encapsulate TMZ in SLN, as a dodecyl (long chain) ester derivative (TMZ-C12) [16]. This approach should allow protecting TMZ from the aqueous environment, slowing down the drug release and the following spontaneous activation, that occurs in the bloodstream at neutral pH. The therapeutic potential of the new formulation was then assessed on human and mouse melanoma cells in in vitro. Finally, the efficacy of TMZ-C12 loaded SLN (SLN-TMZ) was assessed in the mouse B16-F10 melanoma model.

## 2. Results

Blank SLN and SLN-TMZ were obtained through the coacervation technique. Precipitation of sodium behenate from hot micellar solution was obtained by two alternative methods (Table 1). Method 1 was the classic method employed in previous experimental works, and involves the drop-wise addition of sodium phosphate followed by hydrochloric acid [15,16]. In method 2, sodium hydroxide is added to the starting micellar solution, because in hot acqueous solution sodium behenate partially undergoes protonation to insoluble behenic acid, leading to a slight turbidity. After sodium hydroxide addition, a complete and regular precipitation of behenic acid nanoparticles was obtained by substituting sodium phosphate with ammonium chloride.

**Figure 2:**
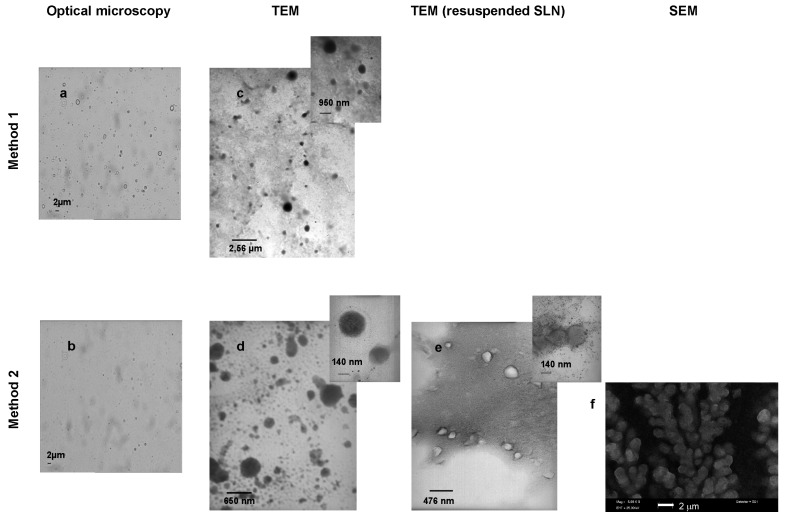
Microscopy of blank SLN. (**a**) optical microscopy of SLN obtained by method 1; (**b**) optical microscopy of SLN obtained by method 2; (**c**) TEM of SLN obtained by method 1; (**d**) TEM of SLN obtained by method 2; (**e**) TEM of SLN, centrifuged and resuspended, obtained by method 2; (**f**) SEM of SLN obtained by method 2.

Through optical microscopy (Figure 2a,b), we noticed that method 2 leads to a more homogeneous nanosuspension, being free from microparticle impurities. This was confirmed by particle size and polydispersity reduction observed in dynamic light scattering (DLS) analysis (Table 2).

Particle shape was investigated through trasmission electron microscopy (TEM) analysis (Figure 2c–e). Microparticle impurities were detectable only in SLN obtained with method 1, while mean particle size was comprised between 200–400 nm with method 2. Indeed, SLN by method 2 were deeper investigated. In fact, PVA9000 removal, obtained by SLN centrifugation and resuspension in distilled water, led to a less contrasted image, and the nanoparticle surface seemed rougher and less regular, compared to non-centrifuged SLN. Thus, an interaction between SLN and PVA9000 at the surface can be hypothesized. This was confirmed by scanning electron microscopy (SEM) (Figure 2f). Here the polymer seems to act as a coating shell around the groups of nanoparticles. However, both TEM and SEM allow to assess a rough, but spherical shape of SLN by method 2, with approximatively the same particle size detected by DLS.

**Figure 3:**
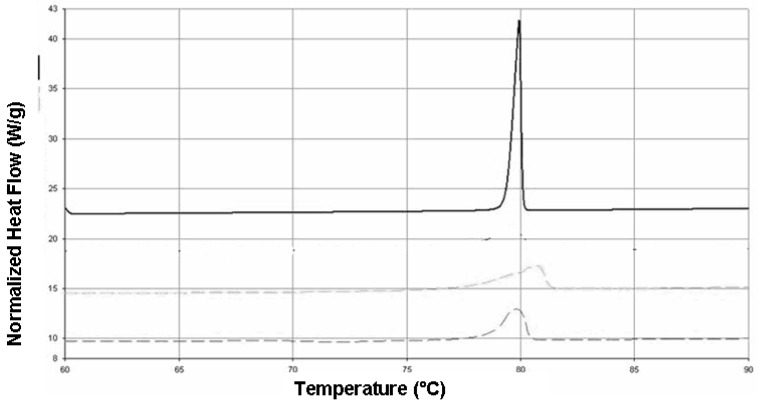
DSC of raw behenic acid (upper curve), blank SLN obtained with method 1 (intermediate curve) and method 2 (lower curve).

Figure 3 shows differential scanning calorimetry (DSC) patterns of raw behenic acid and SLN obtained with method 1 and 2: the nanoparticles showed the endothermic peak of behenic acid, with a reduction of crystallization degree compared to raw material, regardless of the precipitation method employed.

Since SLN obtained with method 2 showed a more reduced and homogeneous particle size, they were employed for the following parts of the study.

TMZ-C12 was loaded in SLN after melting of blank nanoparticles, owing to a method employed in previous works [16,17]. In fact, operating at acid pH does not hamper the stability of the alkylating drug. In order to reach the therapeutic dose, SLN-TMZ were concentrated through ultracentrifugation and resuspension in a small amount of PVA9000/citrate buffer. The pH of the final suspension was kept acid through diluted citrate buffer. In this condition TMZ-C12 was stable during storage. The resuspension process did not compromise either the particle size or the TMZ EE% of the nanoparticles (Table 2). EE% was measured either by centrifugation or by gel filtration, and a lower EE% was obtained by the latter. This could be attributed to the fact that size exclusion requires rather long time and it is performed by employing pH = 7.4 PBS buffer as eluent. In these conditions partial cleavage of TMZ ring can occur.

**Figure 4:**
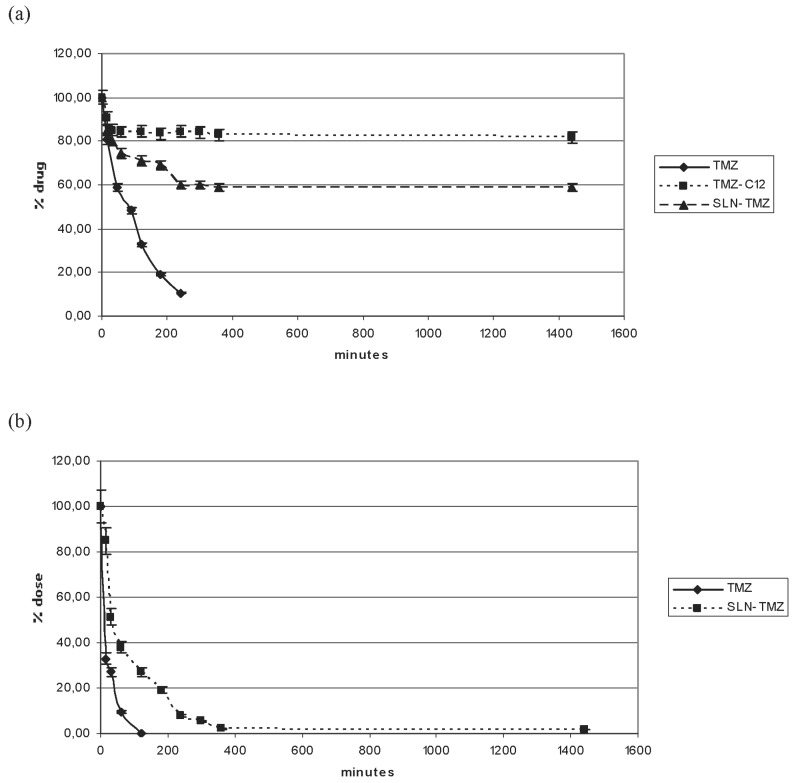
Stability of formulations in RPMI 1640 and in plasma. (**a**) Stability in RPMI 1640 of free TMZ, TMZ-C12 and SLN-TMZ; (**b**) Stability in plasma of free TMZ and SLN-TMZ. Error bar means SEM.

The stability of TMZ and its prodrug loaded in SLN was investigated both in Roswell Park Memorial Institute 1640 medium (RPMI 1640) and in plasma (Figure 4). Both media caused drug degradation, even if this phenomenon was quicker in plasma. In RPMI 1640 the lipophilic prodrug (TMZ-C12) was cleaved more slowly than the parent drug (TMZ), probably because of the influence of its low solubility, and the loading in SLN increased its stability. In the plasma the pure prodrug stability was not investigated because of its low solubility, but important differences were detected between free TMZ and SLN-TMZ, which resulted more stable.

Cytotoxicity studies were performed on different cell lines, employing blank SLN, free TMZ and SLN-TMZ, while pure TMZ-C12 was excluded, because of its reduced solubility, that would make these experiments troublesome. The treatment was performed on human and mouse melanoma cells lines (A2058, JR8 and B16-F10) as potential chemotherapy targets.

**Figure 5:**
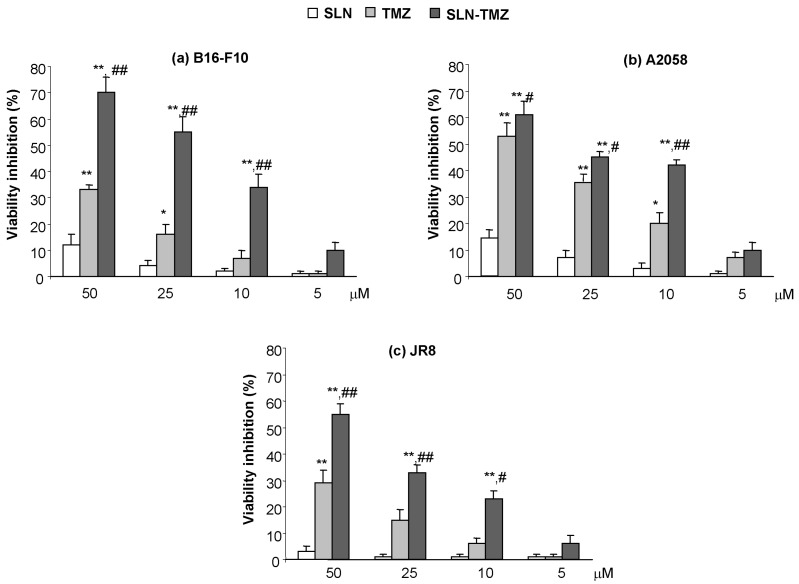
Cytotoxicity on melanoma cells: (**a**) B16-F10; (**b**) A2058; (**c**) JR8. Cells were treated with free TMZ and SLN-TMZ at 50–5 μM concentration for 72 h. Then, the cell proliferation reagent WST-1 is used for 2 h. Cells that had received no drug, as control, were normalized to 100%, and the readings from treated cells were expressed as % of viability inhibition. Eight replicates were used to determine each data point and five different experiments were performed. Data are shown as mean ± SEM. Statistical analyses were performed using one-way ANOVA and the Dunnett test. ** *p* < 0.01 compared to the PBS-treated group. * *p* < 0.05 compared to the PBS-treated group. ## *p* < 0.01 compared to the TMZ-treated group. # *p* < 0.05 compared to the TMZ-treated group.

Figure 5 shows that SLN-TMZ displayed higher toxicity than free TMZ in all melanoma cell lines. Indeed, on B16-F10 cell line, SLN-TMZ induce 70 ± 6% of viability inhibition at 50 μM, while free TMZ only 34 ± 2%. The inhibitory effect was concentration dependent. Thus, at the concentration of 10 μM, while SLN-TMZ caused a 35 ± 4% viability inhibition, free TMZ was ineffective. 

**Figure 6:**
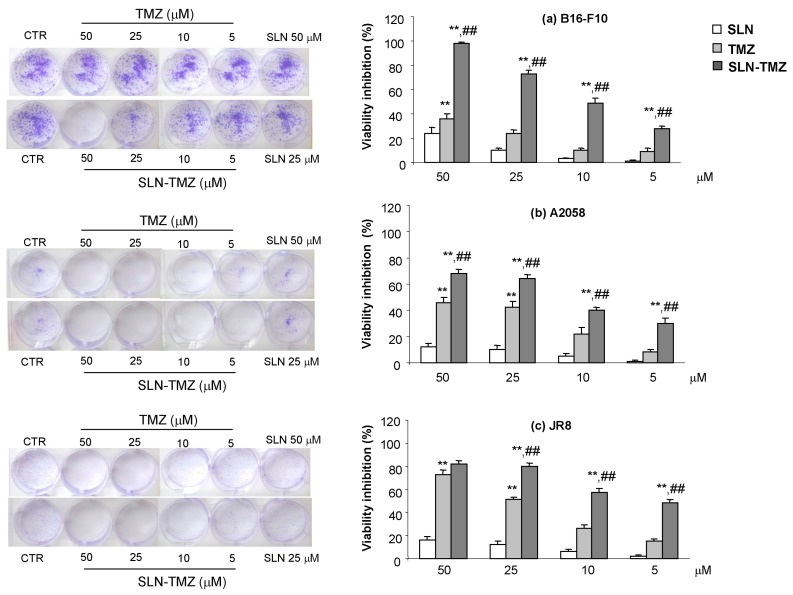
Clonogenic assay: (**a**) B16-F10; (**b**) A2058; (**c**) JR8. Cells were treated with free TMZ and SLN-TMZ at 50–5 μM concentration for 72 h. Then, the cells medium was changed and cells were cultured for additional 7 days in a drug-free medium. Colonies were then photographed. Then, the cells were treated with acetic acid to induce a completely dissolution of the crystal violet and absorbance was evaluated. Five different experiments were performed. Data are shown as mean ± SEM. Statistical analyses were performed using one-way ANOVA and the Dunnett test. ** *p* < 0.01 compared to the PBS-treated group. ## *p* < 0.01 compared to the TMZ-treated group.

In order to validate these findings, clonogenic survival assays were performed. In fact, while 2-(4-iodophenyl)-3-(4-nitrophenyl)-5-(2,4-disulfophenyl)-2H-tetrazolium (WST-1) assay reveals cell viability after drug treatment, clonogenic assay shows only the viable cells that are still able to proliferate, after drug removal by the culture medium. Indeed, cells were treated with the drugs for 72 h, then after the removal of the drug, the cultures were prolonged in drug free medium for the following 7 days, when only a fraction of the seeded cells retained the ability to produce colonies [18]. Results confirmed those obtained with the WST-1 assay (Figure 6). 

**Figure 7:**
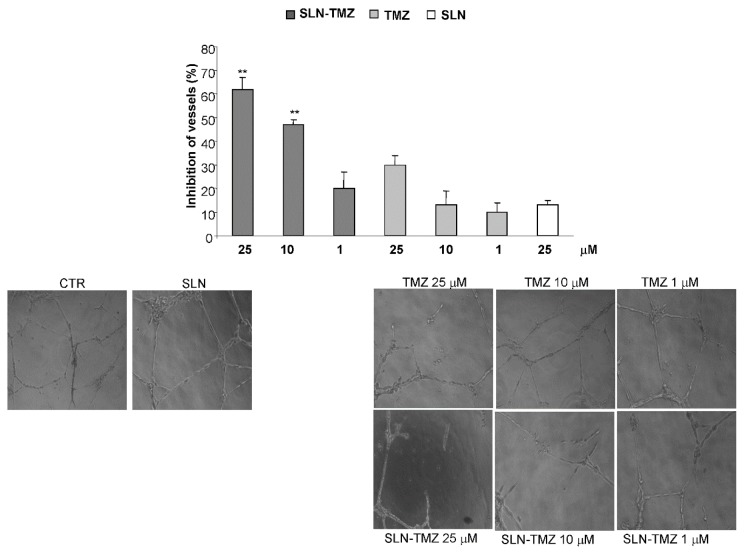
Tube formation assay on HUVEC. Cells were seeded onto 48-well plates (5 × 10^4^/well) previously coated with 75 μL of growth factor-reduced Matrigel, in the presence and in the absence of different concentrations of each drug formulation. Tube formation was then photographed (10× magnification) and evaluated by counting the total number of tubes in three wells; five different experiments were performed. Data are shown as mean ± SEM. Statistical analyses were performed using one-way ANOVA and the Dunnett test. ** *p* < 0.01 compared to the PBS-treated group.

Angiogenesis is essential for tumour growth and metastasization. Kurzen H. et al. (2003) [19] demonstrated that TMZ inhibits angiogenesis when used at low and non-toxic doses. Therefore we compared the anti-angiogenetic effect of SLN-TMZ and TMZ on human umbilical vein endothelial cells (HUVEC) cells. In preliminary experiments, we selected drug concentrations that were not cytotoxic on HUVEC cells after 24 h treatment. Then, we assessed their effects on the tubuli-formation assay in the presence or absence of titrated amounts of each drug formulation. The morphology of capillary-like structures formed by HUVEC was analyzed after 15 h of culture. Results showed that SLN-TMZ significantly inhibited tubuli-formation in a concentration-dependent manner. At 25 μM, the structure and organization of the tubuli were strongly disrupted and at 1–10 μM, only few cells were able to form basic tubuli. By contrast, free TMZ was less effective and significant effect was measured only at the highest 25 μM concentration (Figure 7). The inhibition of capillary network formation was 60 ± 5% and 48 ± 2% for SLN-TMZ 25 and 10 μM, respectively; 30 ± 4% and 15 ± 5% for free TMZ 25 and 10 μM, respectively.

**Figure 8:**
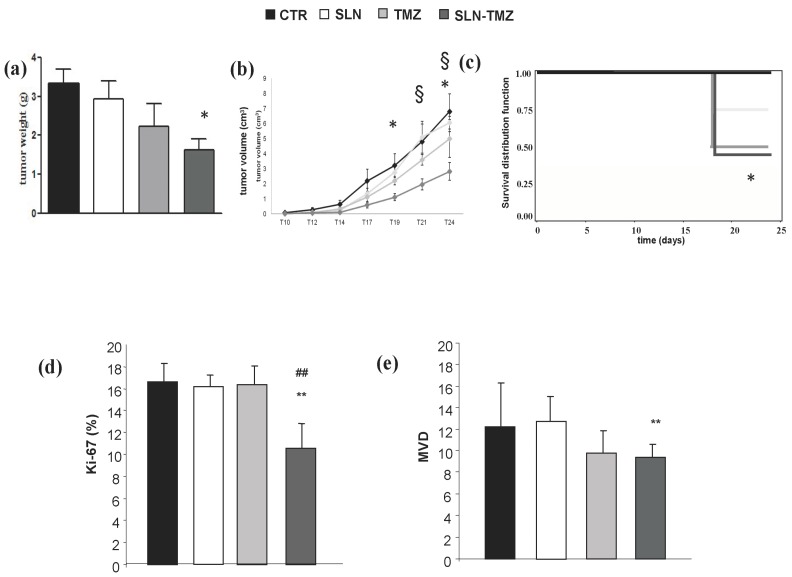
In vivo experiments on mouse melanoma model. C57BL6/J mice were injected subcutaneously with B16-F10 cells (1 × 10^5^ in 100 μL/mouse). Ten days after the tumor injection, mice were treated three times a week for two weeks by i.v. injection of free TMZ, SLN-TMZ or empty SLN (100 μL each–0.5 μM/g) or the same volume of PBS as control. Mice were sacrificed at the end of the experiment. Then, tumors, organs and blood were collected and kept for histological analysis. Graphs show: (**a**) tumor weight (grams—mean ± SEM), (**b**) tumor volume curves (cm^3^—mean ± SEM), (**c**) survival distribution function, (**d**) % of Ki-67-positive cells among tumor cells (**e**) MVD determined by counting the individual CD31+ microvessels. Ten randomly selected areas from three tumors from each group were analyzed; data are expressed as media and interquartile ranges. Statistical analyses were performed using one-way ANOVA and the Dunnett test. * *p* < 0.05; ** *p* < 0.01 compared to the PBS-treated group. § *p* < 0.05 compared to the empty SLN group. ## *p* < 0.001 compared to the TMZ-treated group.

To assess the in vivo effect of our formulations, we compared development of melanoma in C57BL6/J mice using the transplanted B16-F10 model. Tumor measurements, performed after animal sacrifice, showed that SLN-TMZ significantly decreased tumor growth, since tumor weight was inhibited by 50% (Figure 8a). Moreover, all mice treated with SLN-TMZ survived to the end of the experiments, compared to about 50% of control mice (Figure 8b). By contrast, blank SLN did not show any effect on mouse survival and tumor growth, and free TMZ displayed lower or not significant effects. Improved survival rate, compared to controls, could be related to a lower tumor Ki-67 expression in the SLN-TMZ group (Figure 8c). An additional positive effect on SLN-TMZ treatment efficacy, compared to controls, could be ascribed to the anti-angiogenic action of SLM-TMZ (Figure 8d).

**Figure 9:**
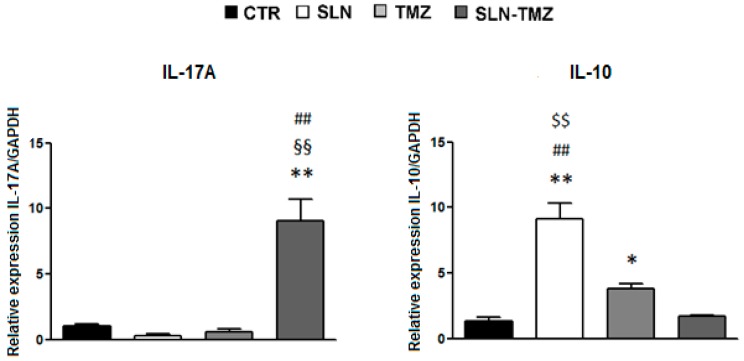
Effect of the different in vivo treatments on IL-17A and IL-10 expression in the tumor mass. IL-17A and IL-10 expression was evaluated in the tumor mass by Real Time PCR analysis. Data are expressed as mean ± SEM of fold increase versus GAPDH expression (*n* = 5). Statistical analyses were performed using one-way ANOVA and the Dunnett test. * *p* < 0.05; ** *p* < 0.01 compared to the PBS-treated group. §§ *p* < 0.01 compared to the empty SLN group. ## *p* < 0.01 compared to the free TMZ-treated group. $$ *p* < 0.01 compared to the SLN-TMZ-treated group.

To assess the effect of the therapies on the anti-tumor-immune response, we assessed expression of IFN-γ and IL-17A marking pro-inflammatory T helper type 1 (TH1) and T helper type 17 (TH17) cells, respectively, and IL-10 marking anti-inflammatory regulatory T cells (Treg) in the tumor mass by real time PCR (Figure 9). Results showed that treatment with SLN-TMZ strikingly increased expression of IL-17A (9.06 ± 1.62) compared to mice treated with PBS (1.04 ± 0.11, *p* < 0.01), or empty SLN (0.29 ± 0.12, *p* < 0.01), or free TMZ (0.59 ± 0.23, *p* < 0.01). By contrast, expression of IL-10 was increased in mice treated with empty SLN (9.14 ± 1.16) compared to mice treated with PBS (1.30 ± 0.35, *p* < 0.01) or free TMZ (3.79 ± 0.35, *p* < 0.01) or SLN-TMZ (1.70 ± 0.08, *p* < 0.01), and in mice treated with free TMZ compared to mice treated with PBS (*p* < 0.05). By contrast, expression of IFN-γ was nor substantially modulated by any treatment.

**Figure 10:**
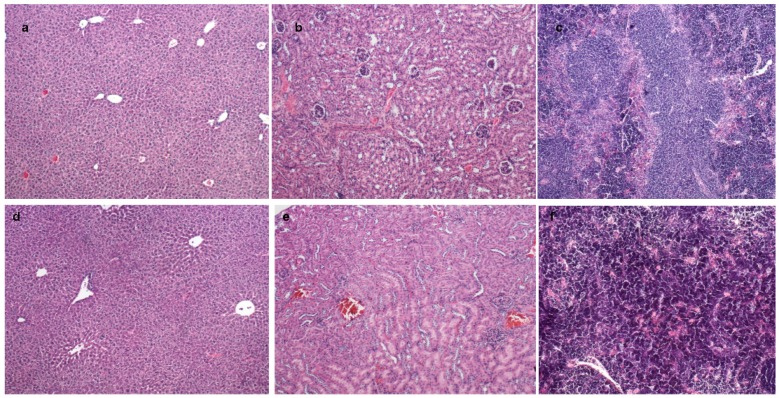
Histopathology of liver (**a**), kidney (**b**) and spleen (**c**) in SLN-TMZ group mice; liver (**d**), kidney (**e**) and spleen (**f**) in control group mice (haematoxylin and eosin, 100×).

SLN-TMZ did not display any apparent toxic effect on mice since it did not affect their weight, feeding behavior and motor activity (data not shown). In line with absence of toxic effects, histological analysis of explanted liver and kidney tissues did not detect any morphological alteration (Figure 10).

**Figure 11:**
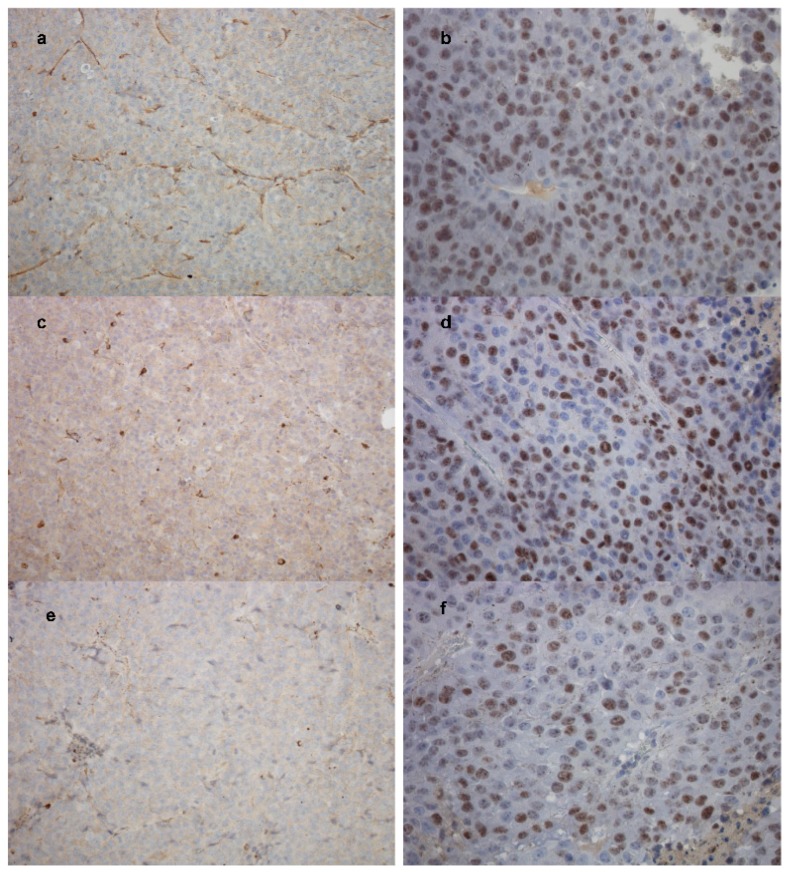
Immunohistochemical staining of CD31 (200×) and Ki-67 (400×) in tumor sections. (**a**,**b**) CD31 and Ki-67 in control group; (**c**,**d**) CD31 and Ki-67 in free TMZ group; (**e**,**f**) CD31 and Ki-67 in SLN-TMZ group.

Immunohistochemical (IHC) analysis of the tumors showed higher Ki-67 staining (marking proliferating cells) in control mice and TMZ-treated mice, compared to SLN-TMZ treated mice (Figure 8d and Figure 11b,d,f). Moreover, to confirm in vitro new vascularization inhibition, tumor angiogenesis was evaluated by tumor microvessel density (MVD) [20] in tumor sections stained for CD31. A decreased CD31 expression was revealed in treated mice (Figure 11c,e) compared to controls (Figure 11a), even if differences between free TMZ and SLN-TMZ groups were not significant (Figure 11e).

## 3. Discussion

TMZ is an alkylating chemotherapeutic drug commonly employed for GB treatment by oral route. Moreover, it has been proposed for the treatment of melanoma via topical application [12]. Its low stability at physiologic pH limits the administration of TMZ via parenteral routes. Delivery and sustained release of TMZ via a nanomedicine formulation could provide a tool both to enhance its therapeutic index. The efficient encapsulation of TMZ in nanoparticle-based systems that can protect the drug from rapid degradation in physiological solutions is a challenge and several carriers of TMZ, including functionalized liposomes, lactoferrin nanoparticles, poly[lactic-co-glycolic acid] (PLGA) nanoparticles, etc. have been tested for their efficacy [21,22,23,24]. The success of these formulations was, however, limited due to: the lack of specific delivery to tumor cells, the poor drug cell uptake, the excessive drug efflux from cells, and the inability to maintain the cytotoxic efficacy.

Our approach is based on lipophilization of the parent molecule and following encapsulation in a nanoparticulate lipid matrix, in order to increase its stability after i.v. administration, which should lead to a an improved pharmacokinetic profile with regard to its therapeutic potential.

To this aim, we chose a feasible technique to produce SLN-TMZ that is fatty acid coacervation. Compared to previous works [15,16], the formulation technique was changed. By employing method 2 a more homogeneous sized of the behenic acid nanosuspension was obtained, as assessed by DLS analysis (Table 2), optical microscopy and TEM (Figure 2a–e). This is likely due to the addition of sodium hydroxide to the starting micellar solution, which inhibits the spontaneous protonation of sodium behenate in hot water. In method 1, this unwanted phenomenon could drive the nucleation of crystals during sodium monohydrogen phosphate/hydrochloric acid addition, leading to a less homogeneous particle size distribution and to the presence of microparticle impurities. When starting pH is set to alkaline conditions with sodium hydroxide, the micellar solution is completely clear, but we have to shift to ammonium chloride/hydrochloric acid to observe the complete precipitation of behenic acid. Regardless of the precipitation method, employed and of the particle size obtained, DSC confirmed that nanoparticles were constituted from solid behenic acid (Figure 3). From SEM and TEM analyses (Figure 2d–f) we can hypothesize that PVA9000, which is employed as a water soluble polymeric stabilizer, effectively interacts with nanoparticles surface, forming a hydrophilic coating on their surface. Encapsulation of TMZ-C12 in the lipid matrix was obtained after melting of blank SLN in order to avoid the risk of TMZ ring cleavage at alkaline pH of the micellar solution. Then SLN were concentrated by ultracentrifugation and resuspension in acid buffer, in order to preserve TMZ ring stability. Sometimes resuspension of SLN can be troublesome, because of particle irreversible aggregation after ultracentrifugation; in our case this process was feasible, because of PVA9000 employed in resuspending medium. Just before in vivo administration, pH of the suspension was adjusted to neutral with sodium carbonate, in order to avoid pain during injection.

Instability of TMZ, both in cell medium and in plasma, can be ascribed to the non-enzymatic ring opening. The collected data on SLN-TMZ level in RPMI 1640 and in plasma allow to predict a changed pharmacokinetic profile for SLN-TMZ compared to free parent drug. This hypothesis was also suggested by results obtained on both tumour cell cultures and animal models, which in addition provided the first evidence on the improved therapeutic potential of the new formulation. In fact, while blank nanoparticles did not exert not toxic effects (Figure 5), SLN-TMZ were more effective against tumor cells, compared to free TMZ. Cytotoxicity was tested on two human melanoma cell lines, as well as on a mouse melanoma cell line, in perspective of employment on an in vivo melanoma mouse models (Figure 5 and Figure 6). The superiority of SLN-TMZ vs. TMZ was shown in vitro. The anti-angiogenic activity of TMZ, which is described in the literature as an important factor to inhibit the tumor growth [19], was also improved with SLN-TMZ (Figure 7). The increased efficacy of the nanoformulation may be related to its improved stability in culture medium (Figure 4a). However, it cannot be ruled out that a different mechanism of cell internalization between SLN-TMZ and TMZ may also play a role.

The in vitro results indicate that the nanoformulated TMZ-C12 is suitable for i.v. administration, because of its increased stability in cell culture medium and plasma, and enhanced activity against tumor cells. Moreover, the improved efficacy shown in vitro allows predicting a reduction of the therapeutic dose in vivo, with decrease of side effects. In fact, in our preliminary in vivo experiments, we used a dosage regime less aggressive than those employed in literature [25,26,27,28]. Results showed that, at these sub-therapeutic doses, free TMZ did not show significant effects on tumor growth and weight compared to control; SLN-TMZ, instead, displayed significant effects on these parameters, and also on mouse survival, which was increased from 50 to 100%. The superiority of SLN-TMZ vs. TMZ was also shown by Ki67 expression in tumor cells, which was significantly decreased only in mice treated with SLN-TMZ. By contrast, despite in vitro tumor angiogenesis was highly inhibited by SLN-TMZ, in vivo similar effects on MVD were obtained by treatment with SLN-TMZ and free TMZ.

The observation that treatment with SLN-TMZ increases expression of IL-17A without affecting expression of IL-10 suggests that this treatment may also exert positive effects on the anti-tumor immune response by increasing the TH17/Treg cell ratio. This marks a difference with treatment with free TMZ that increased expression of IL-10 without affecting expression of IL-17, which suggests that it induced a decrease of the TH17/Treg ratio. The striking effect of empty SLN in increasing expression of IL-10 is in line with previous data obtained in rat cells in vitro [29].

The landscape of current treatment for advanced melanoma has changed rapidly in the last few years, and there are now several different classes of therapy that can be offered to patients depending on their mutational status and disease burden. TMZ is not adopted as a first line therapy for melanoma, especially after the approval of immunotherapeutic drugs, such as ipilimumab and nivolumab [30,31,32,33]. However, only 25% of patients respond to these agents and several of them became resistant. Therefore, investigation of novel strategies for the delivery of new and old drugs could allow achievement of further innovations [34]. Nanotechnology allows to potentially improve the effectiveness of traditional cytotoxic chemotherapeutics, overcoming their drawbacks and side effects. The employment of nanoparticulate TMZ has been recently reported for melanoma treatment. Targeting and uptake of TMZ loaded polyamide-amine dendrimer was demonstrated in A375 metastatic melanoma cell line, with an increase of sensitivity to the drug [35]. Likewise, this work shows that TMZ delivery using SLN may be an effective manner to increase the anti-cancer effect of the drug, allowing for decreasing dosage and, possibly, the side effects. However, further studies are needed to deeply investigate the fate of SLN-TMZ after i.v. administration, with regards to the nanoparticles half-life in the bloodstream and their accumulation in the tumor site, as well as to determine the optimal drug dosage and frequency of administration to increase the therapeutic efficacy in vivo.

## 4. Experimental Section

### 4.1. Materials

#### 4.1.1. Chemicals

Sodium behenate was from Nu-Chek Prep, Inc. (Eleysian, MN, USA); acetic acid, triethylamine, sodium nitrite, anhydrous dimethylformammide (DMF), TMZ, 80% hydrolyzed polyvinyl alcohol of 9000–10,000 MW (PVA9000), penicillin–streptomycin, Hepes, M199 medium, Dulbecco’s Modified Eagle Medium (DMEM), Roswell Park Memorial Institute 1640 medium (RPMI 1640), heparin, and crystal violet were from Sigma-Aldrich (St. Louis, MO, USA); sodium monohydrogen phosphate, citric acid, ammonium chloride, sodium hydroxide from Azienda Chimica e Farmaceutica—ACEF (Fiorenzuola d’Arda, Italy); sulphuric acid, hydrochloric acid from Merck (Darmstadt, Germany); Br-dodecane, dichlorometane, chloroform, methanol and acetonitrile from Carlo Erba (Val De Reuil, France); EBM-2 basal medium was from Lonza (Basel, Switzerland); fetal bovine serum (FBS) Gold from PAA The Cell Culture Company (Pasching, Austria); rat collagen-I was from Trevigen (Gaithersburg, MD, USA); chemically defined lipid concentrate was from Invitrogen Life technologies (Carlsbad, CA, USA); fetal calf serum (FCS) was from Invitrogen (Burlington, ON, Canada); Matrigel, were from BD Biosciences; 2-(4-iodophenyl)-3-(4-nitrophenyl)-5-(2,4-disulfophenyl)-2H-tetrazolium, monosodium salt (WST-1) and 1-methoxy-5-methylphenazinium methylsulfate (1-methoxy-PMS) were from Dojindo Molecular Technologies (Kumamoto, Japan).

Deionized water was obtained by a MilliQ system (Millipore, MO, USA). All other chemicals were analytical grade and used without any further purification.

#### 4.1.2. TMZ-C12 Synthesis

**Figure 12:**
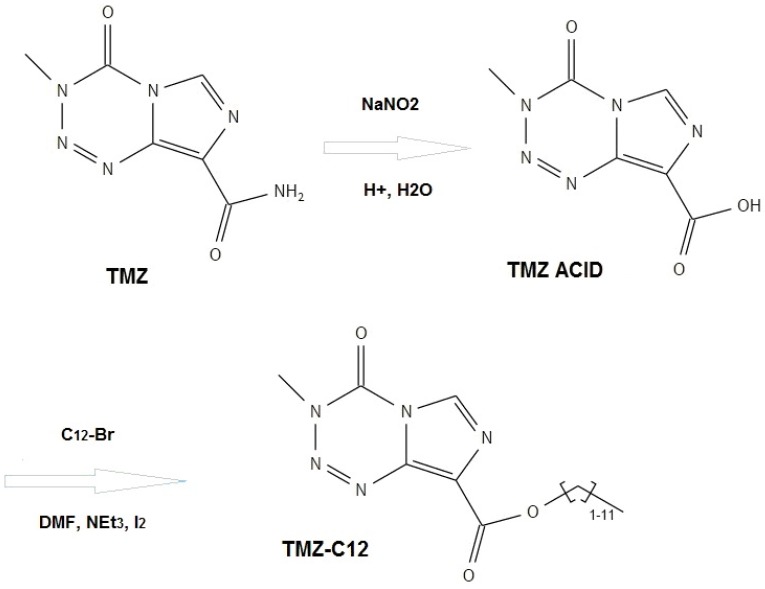
Scheme of TMZ-C12 synthesis.

TMZ-C12 synthesis was performed according to the literature (Figure 12) [12,16].

#### 4.1.3. SLN Preparation

Blank SLN were prepared according to the coacervation method [15], as reported in Table 1. Briefly, sodium behenate was dispersed in water with PVA9000 and the mixture was then heated under stirring (300 rpm), to obtain a clear solution. Two acidifying (coacervating) conditions were compared: sodium monohydrogen phosphate, followed by hydrochloric acid; and ammonium chloride followed by hydrochloric acid; they were added drop-wise to the mixture until complete behenic acid precipitation. The obtained suspension was then cooled under stirring at 300 rpm until 15 °C temperature was reached.

For drug loaded SLN, TMZ-C12 was dissolved in a small amount of DMF, and this solution was added to the blank SLN, led to their melting point; the drug was allowed to partition in the melted lipid for 5 min under stirring and then cooled to room temperature.

For in vitro and in vivo experiments, SLN were concentrated 10-fold under sterile hood, in order to reach a drug therapeutic concentration/dose. 10 mL of suspension were centrifuged at 62,000× *g* (Allegra 64R centrifuge, Beckman Coulter, Brea, CA, USA) for 15 min, followed by re-suspension of the precipitate in 1 mL of 0.01 M citrate buffer pH = 3.0 containing 100 mg/mL PVA9000, with the help of an ultrasonic bath (Transsonic, Elma Schmidbauer GmbH, Singen, Germany). 

#### 4.1.4. SLN Characterization

SLN particle sizes and polydispersity indexes (PDI) were determined one hour after preparation using dynamic light scattering technique-DLS (Brookhaven, NY, USA). Size measurements were obtained at an angle of 90° at 25 °C. All data were determined in triplicate.

The homogeneity of the suspension was checked with optical microscopy (DM2500, Leica Microsystems, Wetzlar, Germany). Particle shape was determined through Transmission Electronic Microscopy (TEM-CH10, Philips, Amsterdam, The Netherlands) and Scanning Electronic Microscopy (SEM-Stereoscan 410, Leica Microsystems, Wetzlar, Germany). For TEM analysis, SLN were employed as such, or after centrifugation and resuspension in water, in order to discriminate for the effect of PVA9000. Instead, SLN were properly diluted (1:25) in order to be analyzed with SEM; then samples were placed on the stub and left to dry under vacuum for one night. The sample obtained, not being conductive, has been subjected to gold metallization by sputtering.

Differential Scanning Calorimetry (DSC) was performed through a DSC7 (Perkin Elmer, Waltham, MS, USA) on SLN centrifuged and dried under vacuum, in order to assess their solid state and the absence of supercooled melts.

Entrapment efficiency (EE%) determination was performed as follows: 0.5 mL SLN suspension was diluted with 0.5 mL water and centrifuged for 15 min at 62,000× *g*; the precipitate was washed with 1 mL acetonitrile/0.1 M pH = 3.0 citrate buffer to eliminate adsorbed drug; the lipophilic prodrug was extracted from the solid residue by dissolution in 0.3 mL dimethylsulfoxide (DMSO) and 0.2 mL acetonitrile, then 0.25 mL 0.1 M citrate buffer pH = 3.0 was added to precipitate the lipid matrix and the supernatant was injected in a Reversed Phase High Performance Liquid Chromatography (RP-HPLC) system (Shimadzu LC-10, Kyoto, Japan). EE% was calculated as the ratio between drug amount in the residue and the weighted one.

EE% was also determined by size exclusion. 1 mL SLN underwent gel filtration using a matrix of cross-linked of agarose (Sepharose CL 4B) as stationary phase. The opalescent fractions containing the purified SLN were concentrated under nitrogen up to 1 mL final volume. The prodrug in the resultant suspension was determined solubilizing 0.05 mL SLN into 0.95 mL acetonitrile and analyzing it by HPLC. In this case, EE% was calculated as the ratio between the drug recovery after and before gel filtration.

#### 4.1.5. Stability Studies in Plasma and Cell Medium

A suitable method for the determination of stability of free TMZ, free TMZ-C12, and SLN-TMZ, both in cell culture medium (RPMI 1640, Sigma-Aldrich, St. Louis, MO, USA) and in rat plasma has been developed. Briefly, stability was assessed by dissolving/suspending under stirring at 37 °C 0.5 mM of TMZ, or TMZ-C12, or SLN-TMZ, alternatively in RPMI 1640, or in rat plasma, in separate experiments. At scheduled times 100 μL were withdrawn and centrifuged at 16,000× *g* for 1 min, and the supernatant was injected in the HPLC system, while the precipitate, was dissolved in 100 μL acetonitrile before injection. Stability of drugs was evaluated by considering the cumulative amount in the supernatant and in the precipitate, compared to the starting.

#### 4.1.6. High Performance Liquid Chromatography (HPLC)

TMZ: a reversed-phase column (Mediterranea Sea, 18 5 μm 25 × 0.46 mm) was used. Linear gradient (10 min) from 100% acetic acid to 50% acetonitrile, followed by 5 min at 50% acetonitrile was performed at a flow rate of 1 mL/min. UV-Vis detector was set at 329 nm. Retention time was 9 min.

TMZ-C12: a reversed-phase column (Allsphere™ ODS, 2.5 μm 250 × 4.6 mm) was used. HPLC grade acetonitrile/water (70/30 *v*/*v*) was used as a mobile phase with a flow rate of 1 mL/min. UV-Vis detector was set at 329 nm. Retention time was 8.5 min.

#### 4.1.7. Cytotoxicity Assays 

JR8 human melanoma cells were a kind gift of Dr. Pistoia (Gaslini Institute, Genoa, Italy), A2058 human melanoma cells and B16-F10 mouse melanoma cells were from the American Type Culture Collection (ATCC; Manassas, VA, USA). A2058 was chosen as a tumorigenic and metastatic model cell line [36], while JR8 was chosen as a tumorigenic model cell line [37]. Both are derived from human lymph nodes metastasis. A2058 and JR8 cell lines were cultured in RPMI 1640 and B16-F10 cell lines in DMEM with 10% FBS, 2 mmol/L L-glutamine and penicillin/streptomycin (100 units/mL), at 37 °C in 5% CO_2_ humidified atmosphere. Cells (1 × 10^3^/well) were seeded in 96-well plates and incubated for 24 h. Then, they were treated with at 5–50 μM concentrations of the studied drugs for 72 h. The cell proliferation reagent WST-1 was used, as described by the manufacturer’s protocol. Cells that had received no drug, as control, were normalized to 100%, and the readings from treated cells were expressed as % of viability inhibition. Eight replicates were used to determine each data point and five different experiments were performed. 

#### 4.1.8. Clonogenic Assay

Melanoma cells (8 × 10^2^/well) were seeded into six-well plates. The day after they were treated with different concentrations of the studied drugs for 72 h. Then the medium was changed and cells were cultured for additional 7 days in a drug-free medium. Subsequently, cells were fixed and stained with a solution of 80% crystal violet and 20% methanol. Colonies were then photographed. Then the cells were perfectly washed and 30% *v*/*v* acetic acid was added to induce a completely dissolution of the crystal violet. Absorbance was recorded at 595 nm by a 96-well-plate ELISA reader. Five different experiments were performed.

#### 4.1.9. Tubule-Formation Assay on Human Umbilical Vein Endothelial Cells (HUVEC)

HUVEC were isolated from human umbilical veins by trypsin treatment (1%) and cultured in M199 medium with the addition of 20% FCS, 100 U/mL penicillin, 100 μg/mL streptomycin, 5 UI/mL heparin, 12 μg/mL bovine brain extract, and 200 mM glutamine. The HUVEC were grown to confluence in flasks and used at the 2nd–5th passages. The use of HUVEC was approved by the Ethics Committee of the “Presidio Ospedaliero Martini” of Turin and conducted in accordance with the Declaration of Helsinki. Written informed consent was obtained from all donors. HUVEC were seeded in 96-well plates and treated at 37 °C, 5% CO_2_, for 24 h with different concentrations of the studied drugs. The cell proliferation reagent WST-1 was used. Drug concentrations that were not cytotoxic were used for the tubule-formation assay. Then, HUVEC were seeded onto 48-well plates (5 × 10^4^/well) previously coated with 75 μL of growth factor-reduced Matrigel, in the absence or presence of free TMZ and SLN-TMZ (1–25 μg/mL), or empty SLN at the concentration corresponding to that used with entrapped drugs. The morphology of the capillary-like structures formed by the HUVECs was analyzed by an inverted microscope after 15 h of culture, and photographed with a digital camera. Tube formation was analyzed with an imaging system (Image Pro Plus Software for microimaging, Media Cybernetics, version 5.0, Bethesda, MD, USA). Tubule-formatio was evaluated by counting the total number of tubes in three wells and five different experiments were performed, as previously described [18]. Cells that had received no drug, as control, were normalized to 100% of new formed vessels and the readings from treated cells were expressed as % of vessel inhibition.

### 4.2. Animal Studies

Female 6- to 9-week-old C57BL6/J (The Jackson Laboratory, Bar Harbor, ME, USA) mice were bred under pathogen-free conditions in the animal facility of the University of Eastern Piedmont, and treated in accordance with the University Ethical Committee and European guidelines. The mice were injected subcutaneously with B16-F10 cells (1 × 10^5^ in 100 μL/mouse) and the tumour growth was monitored every two days. Ten days after the tumor induction, the mice were treated via the i.v. injection of TMZ, SLN-TMZ or empty SLN (100 μL each—0.5 μmol TMZ/g) or the same volume of PBS as control. Since all the formulations were obtained in diluted acid buffers, in order to preserve TMZ ring stability, prior to animal administration pH of the suspension was neutralized with 10 μL of 0.2 M Na_2_CO_3_. The treatment had be carried out three times a week for two weeks (6 i.v./mouse) and the mice were sacrificed after three days after the last injection, or when they displayed sufferance. Six animals for groups were employed for each group.

#### 4.2.1. Histology and Immunohistochemistry on Animal Specimens

After euthanasia, all animals underwent complete necropsy. Lung, liver, kidney and spleen were collected and stored in 10% neutral buffered formalin for histological evaluation. Samples were trimmed into cassettes, paraffinized, sectioned (5 μm thick), stained with haematoxylin and eosin, and evaluated by light microscopy. 

Immunohistochemical staining was performed on sections of selected tumors. Primary antibodies included a monoclonal antibody Ki-67 (1:100 dilution; code M7248; Dako, Santa Clara, CA, USA) and a polyclonal anti-CD31 (1:100 dilution; code ab28364; Abcam, Cambridge, UK). Antibodies were detected using the avidin–biotin–peroxidase complex technique with the Vectastain ABC-AP Kit (Universal; Vector Laboratoires, Burlingame, CA, USA). Antigen retrieval was done by heating the sections in citrate buffer (0.01 M, pH 6.0) at 98 °C for 25 min. Endogenous peroxidase activity was quenched. The slides were then incubated overnight in a humidified chamber at 4 °C with the primary antibodies, followed by sequential 10 min incubation with biotinylated link antibody and peroxidase labelled streptavidin. The reaction was visualized using 3,3′-diaminobenzidine tetrahydrochloride (Sigma-Aldrich). The nuclei were counterstained with haematoxylin and eosin stain. Positive and negative immunohistochemistry controls were routinely used. The reproducibility of the staining was confirmed by reimmunostaining via the same method in multiple, randomly selected specimens.

All reactions were visualized by light microscopy and assessed blinded by two observers and the discordant cases were reviewed at a multi-head microscope until a consensus was reached. Each slide for histological staining was captured with a Nikon DS-Fi1 digital camera (Nikon, Shinjuku, Japan) coupled to a Zeiss Axiophot microscope (Zeiss, Oberkochen, Germany) using a 40× objective lens. NIS-Elements F software (V4.30.01, Nikon, Shinjuku, Japan) was used for image capturing. Each immunohistochemical marker was evaluated in at least ten different fields, and particularly measuring the numbers of positive cells for cleaved Ki-67 (Image Pro Plus analysis system—Media Cybernetics, version 5.0, Bethesda, MD, USA) and of microvessels for anti-CD31.

#### 4.2.2. Real Time PCR on Tumors

Total RNA was isolated from tumors, using TRIzol reagent (Sigma-Aldrich). RNA (1 μg) was retrotranscribed using the QuantiTect Reverse Transcription Kit (Qiagen, Hilden, Germany). IFN-γ, IL-17A and IL-10 expression were evaluated with a gene expression assay (Assay-on Demand; Applied Biosystems, Foster City, CA, USA). The GAPDH gene was used to normalize the cDNA amounts. Real-time PCR was performed using the CFX96 System (Bio-Rad Laboratories, Hercules, CA, USA) in duplicate for each sample in a 10 μL final volume containing 1 μL of diluted cDNA, 5 μL of TaqMan Universal PCR Master Mix (Applied Biosystems, Foster City, CA, USA), and 0.5 μL of Assay-on Demand mix. The results were analyzed with a ΔΔ threshold cycle method.

#### 4.2.3. Statistical Analysis

Data are shown as mean ± SEM. Statistical analyses were performed with Prism 3.0 software (GraphPad Software, La Jolla, CA, USA) using one-way ANOVA and the Dunnett test. Kaplan-Mayer survival curves were evaluated with the Chi-Square test.

## Figures and Tables

**Table 1:** SLN composition.

	Method 1	Method 2
80% Hydrolyzed polyvinyl alcohol of 9000–10,000 MW (PVA9000)	200 mg	200 mg
Sodium behenate	100 mg	100 mg
NaOH 1 M		120 μL
Na_2_HPO_4_ 1 M	200 μL	
NH_4_Cl 5 M		260 μL
HCl 1 M	400 μL	400 μL
TMZ-C12		4 mg in 400 μL dimethylformammide (DMF)
Deionized Water		10 mL

**Table 2:** SLN particle size and encapsulation efficiency (EE%).

	Particle Size	Polydispersity	EE% Efficiency (Centrifugation)	EE% Efficiency (Gel Filtration)
Blank SLN method 1	400.1 ± 65 nm	0.269 ± 0.83	-	-
Blank SLN method 2	278.6 ± 4 nm	0.066 ± 0.01	-	-
Concentrated blank SLN	278.8 ± 28 nm	0.052 ± 0.02	-	-
SLN-TMZ	279.0 ± 50 nm	0.038 ± 0.01	93.10 ± 3.29	57.91 ± 21.70
Concentrated SLN-TMZ	273.15 ± 5 nm	0.084 ± 0.01	91.10 ± 0.22	N.D.
